# Cabozantinib overcomes ROS1 L2086F NSCLC resistance to lorlatinib: A case report

**DOI:** 10.1097/MD.0000000000043751

**Published:** 2025-08-15

**Authors:** Xunqi Liu, Qiong Chen, Ling Shao, Pu Luo, Jiang Hong An

**Affiliations:** a The Third People’s Hospital of Shenzhen, Second Affiliated Hospital of Southern University of Science and Technology, Shenzhen City, Guangdong Province, China.

**Keywords:** cabozantinib, lorlatinib, ROS1 fusion mutation

## Abstract

**Rationale:**

ROS1 rearrangement a distinct molecular subtype of non-small cell lung cancer that is amenable to targeted therapeutic interventions. Despite the availability of effective targeted therapies, the development of acquired resistance to later-line inhibitors, particularly lorlatinib, remains an inevitable, and clinically significant challenge. The emergence of the ROS1 L2086F mutation as a mechanism of lorlatinib resistance further complicates treatment, with limited therapeutic options available post-resistance. This case report evaluates the potential efficacy of cabozantinib, a multi-targeted kinase inhibitor, as a salvage therapy in this specific and challenging clinical context.

**Patient concerns:**

A 63-year-old, nonsmoking female presented with cough and expectoration.

**Diagnoses:**

The patient was diagnosed with left lower lobe lung adenocarcinoma (cT2N2M1, stage IV), characterized by a CD74-ROS1 fusion mutation. Following disease progression on lorlatinib, next-generation sequencing analysis of pleural effusion identified an acquired ROS1 L2086F resistance mutation, accompanied by a concurrent mTOR mutation and FGFR3 gene amplification.

**Interventions:**

The patient underwent sequential targeted therapy with first-line crizotinib, second-line lorlatinib, and third-line cabozantinib.

**Outcomes:**

The patient demonstrated a progression-free survival (PFS) of 47 months with first-line crizotinib (PFS1) and 11 months with second-line lorlatinib (PFS2). Upon detection of the L2086F mutation, third-line cabozantinib achieved a clinically significant PFS of 12 months (PFS3), accompanied by disease stabilization and symptomatic relief. The overall survival from initial diagnosis was 73 months.

**Lessons:**

This case highlights the clinical efficacy of cabozantinib in overcoming lorlatinib resistance mediated by the ROS1 L2086F mutation, resulting in durable clinical benefits surpassing those of conventional chemotherapy. These findings emphasize the critical role of repeated, comprehensive genomic profiling in managing refractory ROS1-positive non-small cell lung cancer and establish cabozantinib as a viable therapeutic option in this specific resistance context.

## 1. Introduction

ROS1 fusion mutations represent a distinct molecular subtype of non-small cell lung cancer (NSCLC), accounting for approximately 1% to 2% of all cases, with a higher incidence observed among younger, nonsmoking patients exhibiting adenocarcinoma histology.^[1]^ Current international guidelines, including those from the National Comprehensive Cancer Network, recommend first-line therapeutic agents such as crizotinib, entrectinib, repotrectinib, and ceritinib for advanced ROS1-positive NSCLC.^[1]^ Lorlatinib, a potent third-generation brain-penetrant dual ALK/ROS1 inhibitor, is typically employed following the failure of first-generation tyrosine kinase inhibitors (TKIs) like crizotinib.^[2]^ Nevertheless, the development of secondary resistance to lorlatinib poses a significant clinical challenge, with the underlying mechanisms remaining incompletely elucidated. In this context, cabozantinib, a multi-targeted TKI, has demonstrated potential in preclinical studies and emerging clinical observations for addressing specific ROS1 resistance mutations, including L2086F,^[3,4]^ garnering considerable attention within the medical community. This report presents a detailed case of an NSCLC patient with an acquired ROS1 L2086F resistance mutation who achieved substantial clinical benefit from cabozantinib, aiming to contribute valuable insights and evidence to this complex clinical scenario.

## 2. Case presentation

A 63-year-old Chinese female, a never-smoker, presented in September 2018 with chief complaints of persistent cough and expectoration. Physical examination revealed diminished breath sounds in the lower left lung. She had no significant past medical history or family history of cancer. Initial imaging, including a chest computed tomography scan, revealed a mass in the left lower lobe, multiple ground glass nodules in the right lung, and multiple enlarged mediastinal lymph nodes. A brain magnetic resonance imaging scan showed no evidence of distant metastases. A lung puncture biopsy confirmed the diagnosis of left lower lobe adenocarcinoma clinically staged as cT2N2M1, Stage IV. Comprehensive molecular profiling of the tumor tissue was conducted on September 5 using the United States Clinical Laboratory Improvement Amendments (CLIA) certified Burning Rock Biotech Lanclear™168-gene panel, which covers mutations, rearrangements, copy number variations, and fusions. The analysis identified a CD74-ROS1 (C6:R33) fusion mutation, along with CTNNB1 mutation (p.Thr41Ala) and APC (p.Glu601fs) mutations. The patient’s complete clinical course, molecular evolution, and treatment outcomes are summarized in Table 1.

**Table 1:** Timeline of clinical, molecular, and therapeutic events.

Date	Clinical event	Treatment administered	Best response	Progression-free survival (PFS)	Key molecular findings	Notable adverse events
September 2018	Initial diagnosis	Crizotinib	Near complete remission (near CR)	PFS1: 47 mo	Baseline: CD74-ROS1, CTNNB1 p.T41A, APC p.E601fs	Renal cysts
August 2022	Progression disease 1	Lorlatinib	Partial remission (PR)	PFS2: 11 mo	Plasma ctDNA negative	Hypercholesterolemia, cognitive decline
September 2023	Progression disease 2	Cabozantinib (40 mg qd)	Stable disease (SD)	PFS3: 12 mo	Pleural effusion NGS: CD74-ROS1, ROS1L2086F, mTOR p.E2419K, FGFR3 amplification	Palpitations
October 2024	Progression disease 3/death	–	–	–	Not available	–
Total	–	–	–	Overall survival (OS): 73 mo	–	–

APC = adenomatous polyposis coli, CD = cluster of differentiation, CR = complete remission, ctDNA = circulating tumor DNA, CTNNB = catenin beta, FGFR = fibroblast growth factor receptor, mTOR = mechanistic target of rapamycin, NGS = next-generation sequencing, OS = overall survival, PD = progression disease, PFS = progression-free survival, PR = partial remission, ROS = proto-oncogene receptor tyrosine kinase, SD = stable disease.

**Figure 1.**
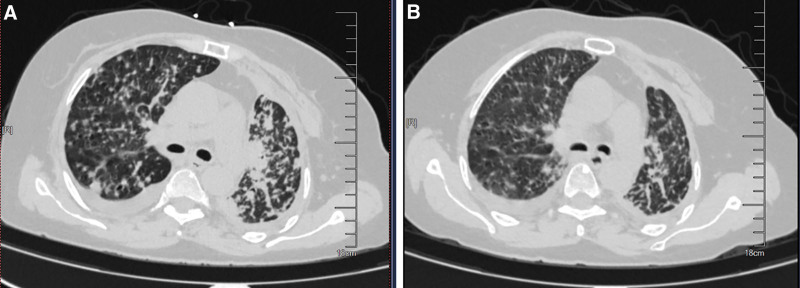
The temporal progression of lorlatinib resistance and the subsequent therapeutic response to cabozantinib were evaluated over a 4-mo period. (A) Initial axial CT imaging performed on September 20, 2023, demonstrated the presence of bilateral pulmonary metastatic lesions, consistent with disease progression and the development of acquired resistance to lorlatinib. (B) Follow-up axial CT imaging conducted on February 2, 2024, after 4 mo of cabozantinib treatment, revealed a marked reduction in both the size and density of multiple metastatic nodules within the bilateral lung fields, with only minimal residual bilateral pleural effusion observed. CT = computed tomography.

The patient initially received first-line crizotinib therapy, attaining near-complete remission with a progression-free survival (PFS1) of 47 months. During this period, renal cysts developed, and whole-brain radiotherapy (PTV 30.0 Gy/3.0 Gy/10 fractions) was administered for brain metastases. In August 2022, the patient presented with dyspnea, and imaging confirmed disease progression. Subsequent next-generation sequencing (NGS) analysis of plasma circulating tumor DNA (ctDNA) on September 27, 2022, failed to detect the CD74-ROS1 fusion mutation. Second-line lorlatinib therapy was initiated, resulting in partial remission with a PFS2 of 11 months. Adverse effects included hypercholesterolemia and cognitive decline, as evidenced by Mini-Mental State Examination and Montreal Cognitive Assessment scores of 20 and 14, respectively. In September 2023, further disease progression was observed. NGS analysis of pleural effusion revealed a complex resistance profile, including the original CD74-ROS1 fusion, an acquired ROS1 L2086F resistance mutation, an mTOR (p.E2419K) mutation, and FGFR3 gene amplification. Third-line cabozantinib therapy was initiated to target the ROS1 L2086F mutation (Fig. 1A). After 4 months, dyspnea symptoms significantly improved, clinical condition stabilized, and follow-up chest computed tomography demonstrated a marked reduction in pulmonary metastases (Fig. 1B). Palpitations, the primary adverse event, were managed through dose reduction. The patient progressed after 12 months of cabozantinib treatment and passed away on October 6, 2024, with a PFS3 of 12 months and a total overall survival (OS) of 73 months from initial diagnosis.

## 3. Discussion

This case illustrates the clinical course of a ROS1-positive NSCLC patient over 73 months, highlighting sequential treatment with 3 generations of targeted therapies and providing valuable dynamic genomic insights into TKI resistance mechanisms.

## 4. Clinical challenges and prognosis in later-line ROS1-positive NSCLC

For ROS1-positive NSCLC patients who have failed multiple lines of TKI therapy, the prognosis remains poor, with limited therapeutic options. Lorlatinib, as a second- or later-line treatment, demonstrates promising efficacy in some patients, though median OS remains limited. A multicenter, open-label, single-arm, phase 1 to 2 trial reported a 35% objective response rate and a median PFS of 8.5 months in crizotinib-pretreated ROS1-positive NSCLC patients receiving later-line lorlatinib.^[2]^ Similarly, a multicenter retrospective analysis of second- or later-line lorlatinib treatment in ROS1-positive NSCLC patients documented an 85.7% response rate and a median OS of 20.0 months from lorlatinib initiation.^[5]^ The 12-month PFS observed with cabozantinib in our patient following lorlatinib failure is notably superior to published data, significantly exceeding the expected survival benefit in this heavily pretreated, refractory patient population. This outcome underscores the clinical relevance of this case and positions cabozantinib as a highly promising therapeutic option.

## 5. Mechanism and efficacy of cabozantinib in overcoming ROS1 L2086F resistance

The ROS1 L2086F mutation represents a critical mechanism of lorlatinib resistance. This mutation, characterized by the substitution of leucine with phenylalanine at amino acid 17 in the solvent-front region of the kinase, impedes the binding of type I TKIs such as lorlatinib. However, it retains sensitivity to type II TKIs, including cabozantinib, as well as type I FLT3 inhibitors like gilteritinib.^[4]^ While gilteritinib has demonstrated efficacy against both wild type and L2086F-mutated ROS1, it is ineffective against the G2032R mutation. Cabozantinib, a type II TKI, employs a distinct binding mode that accommodates this conformational change, thereby effectively inhibiting the kinase activity of the L2086F mutant. This mechanism has been corroborated in preclinical models and multiple case reports, which highlight cabozantinib’s activity against L2086F and other common resistance mutations such as G2032R.^[6]^

To objectively assess the efficacy of cabozantinib in this context, we compared it with the standard-of-care treatment following TKI failure – chemotherapy Table 2. As indicated in Table 2, standard later-line chemotherapy (pemetrexed or docetaxel) after TKI failure typically provides a median PFS of approximately 3 to 5 months.^[7]^ Although some retrospective studies^[8,9]^ suggest that pemetrexed may exhibit enhanced efficacy in ROS1-positive patients, the reported PFS remains substantially lower than the 12 months observed in our case. The 12-month PFS achieved with cabozantinib in this patient, following lorlatinib resistance mediated by the L2086F mutation, is clinically significant and compares favorably to the anticipated outcomes in this refractory setting, where therapeutic options are limited and often exhibit reduced efficacy compared to conventional chemotherapy. This further emphasizes the value of this case report. Additional investigation is warranted to establish overall survival benchmarks following lorlatinib resistance and to evaluate the long-term therapeutic impact of agents such as cabozantinib in this specific patient population.

**Table 2:** Efficacy comparison of later-line treatment options for TKI-pretreated advanced NSCLC.

Therapy	Line of therapy	Median PFS (mo)	Objective response rate (ORR, %)	References
Cabozantinib	Third-line	12.0	Stable disease	This manuscript
Pemetrexed-based chemotherapy	Second-line+	3–8	9–60	^[7,9]^
Docetaxel-based chemotherapy	Second-line+	3–5	9–20	^[1,7]^

Chemotherapy data are derived from studies in previously treated advanced NSCLC patients.

NSCLC = non-small cell lung cancer, ORR = objective response rate, PFS = progression-free survival, TKI = tyrosine kinase inhibitor.

## 6. The potential role of co-occurring genetic alterations

The most thought-provoking aspect of this case is its complex and dynamically evolving genomic landscape. We hypothesize that the patient’s resistance was not solely driven by the ROS1 L2086F mutation but was a multifactorial event involving baseline pathway abnormalities and acquired bypass track activation.The clinical significance of co-occurring mutations in NSCLC remains an active area of investigation.

### Baseline activation of the Wnt/β-catenin pathway:

At initial diagnosis, the patient exhibited concurrent CTNNB1 (p.Thr41Ala) and APC (p.Glu601fs) mutations. CTNNB1, which encodes β-catenin, is known to activate the Wnt signaling pathway upon mutation. Emerging evidence suggests that CTNNB1 mutations may correlate with poor prognosis and therapeutic resistance in lung adenocarcinoma.^[10–12]^ Furthermore, the tumor suppressor gene APC, whose loss of function has been associated with immune checkpoint inhibitor resistance in NSCLC,^[13]^ may also modulate the efficacy of TKI in ROS1-positive NSCLC. We hypothesize that the baseline activation of the Wnt pathway in this patient’s tumor may have established a biological milieu conducive to the subsequent development of TKI resistance.

### Acquired bypass pathway activation:

Following disease progression on lorlatinib, NGS revealed not only the on-target ROS1 L2086F mutation but also an mTOR (p.E2419K) mutation and FGFR3 gene amplification. The mTOR pathway, a critical regulator of diverse cellular processes including growth, survival, proliferation, metabolism, apoptosis, invasion, and angiogenesis, is frequently dysregulated in various malignancies, including NSCLC.^[14]^ The identification of mTOR mutations post-lorlatinib treatment suggests a potential resistance mechanism mediated through activation of the PI3K/AKT/mTOR pathway.^[15–17]^ FGFR3 amplification is another classic bypass activation mechanism for TKI resistance.Aberrations in the FGFR pathway, encompassing both amplifications and fusions, have been demonstrated to promote tumorigenesis and mediate resistance to EGFR TKIs in NSCLC.^[18,19]^ The emergence of these co-alterations, particularly mTOR mutations and FGFR3 amplifications during advanced disease stages, likely contributes to the intricate resistance landscape and disease progression. As evidenced in this study, comprehensive genomic profiling across multiple time points is crucial for identifying concurrent driver mutations and resistance mechanisms that may necessitate targeted therapeutic interventions.

### Implications for cabozantinib’s efficacy:

This multifaceted resistance profile may elucidate its sustained clinical benefit of 12 months. Cabozantinib, a multi-targeted TKI rather than a selective ROS1 inhibitor, exhibits inhibitory activity against mesenchymal-epithelial transition (MET), vascular endothelial growth factor-2, proto-oncogene tyrosine-protein kinase receptor, ROS1, and AXL receptor tyrosine kinase.^[20,21]^ Clinical case reports have documented its efficacy against the L2086F mutation.^[22]^ including a notable instance where a patient with ROS1-rearranged NSCLC, having developed L2086F and F2004V mutations following lorlatinib treatment, demonstrated clinical response to cabozantinib.^[23]^ The established signaling crosstalk between the MET/AXL receptor tyrosine kinase and Wnt/β-catenin pathways further supports the therapeutic rationale. We hypothesize that cabozantinib’s enhanced efficacy may derive from its multi-targeted mechanism: direct inhibition of ROS1 L2086F coupled with concurrent suppression of potential bypass signaling pathways mediated by MET and rat sarcoma-mitogen-activated protein kinase,^[6]^ This comprehensive blockade of tumor escape routes may account for its superior and more durable clinical benefit compared to single-target inhibitors.

## 7. Limitations

This study is subject to several inherent limitations. Firstly, as a single case report, the findings possess limited generalizability, and causal relationships cannot be established; these findings require validation through larger-scale rigorously designed clinical studies. Secondly, although multiple concurrent genetic alterations were identified, the absence of in vitro or in vivo functional experiments precludes precise quantification of the specific contribution of each alteration to the resistance phenotype. Lastly, the unavailability of post-cabozantinib tissue or liquid biopsy samples hindered the investigation of the ultimate molecular mechanisms of resistance to cabozantinib.

Future research should prioritize multicenter, large-scale prospective clinical trials to further evaluate the efficacy and safety of cabozantinib, as well as to elucidate the specific molecular mechanisms by which it overcome resistance. However, it is important to note that cabozantinib therapy is associated with certain adverse effects. In this particular case, the primary adverse event was palpitations, which were effectively managed following dose reduction. This underscores the necessity of vigilant monitoring for adverse reactions in clinical practice and the importance of individualized dose adjustments to optimize both the safety and therapeutic efficacy of treatment regimen.

## 8. Conclusion

This case report clearly demonstrates that cabozantinib achieved a durable clinical benefit of 12 months in a patient with advanced NSCLC who developed resistance to lorlatinib mediated by the *ROS1 L2086F* mutation.This outcome significantly exceeds the expected survival in this refractory patient population and provides important clinical insights.

## Author contributions

**Conceptualization:** Xunqi Liu, Qiong Chen.

**Data curation:** Ling Shao, Pu Luo.

**Formal analysis:** Ling Shao, Pu Luo.

**Writing** – **original draft:** Xunqi Liu, Qiong Chen.

**Writing** – **review & editing:** Jiang Hong An.
